# Spectrum of antibacterial activity and mode of action of a novel tris-stilbene bacteriostatic compound

**DOI:** 10.1038/s41598-018-25080-w

**Published:** 2018-05-02

**Authors:** Nikki Y. T. Man, Daniel R. Knight, Scott G. Stewart, Allan J. McKinley, Thomas V. Riley, Katherine A. Hammer

**Affiliations:** 1 Chemistry, School of Molecular Sciences, The University of Western Australia, Crawley, WA 6009 Australia; 2 School of Biomedical Sciences, The University of Western Australia, Crawley, WA 6009 Australia; 3 PathWest Laboratory Medicine WA, Queen Elizabeth II Medical Centre, Nedlands, WA 6009 Australia; 4 School of Medical and Health Sciences, Edith Cowan University, Joondalup, WA 6027 Australia

## Abstract

The spectrum of activity and mode of action of a novel antibacterial agent, **135C**, was investigated using a range of microbiological and genomic approaches. Compound **135C** was active against Gram-positive bacteria with MICs for *Staphylococcus aureus* ranging from 0.12–0.5 μg/ml. It was largely inactive against Gram-negative bacteria. The compound showed bacteriostatic activity in time-kill studies and did not elicit bacterial cell leakage or cell lysis. Checkerboard assays showed no synergy or antagonism when **135C** was combined with a range of other antibacterials. Multi-step serial passage of four *S. aureus* isolates with increasing concentrations of **135C** showed that resistance developed rapidly and was stable after drug-free passages. Minor differences in the fitness of **135C**-resistant strains and parent wildtypes were evident by growth curves, but **135C**-resistant strains did not show cross-resistance to other antibacterial agents. Genomic comparison of resistant and wildtype parent strains showed changes in genes encoding cell wall teichoic acids. **135C** shows promising activity against Gram-positive bacteria but is currently limited by the rapid resistance development. Further studies are required to investigate the effects on cell wall teichoic acids and to determine whether the issue of resistance development can be overcome.

## Introduction

Antimicrobial resistance (AMR) is one of the most problematic public health issues that society faces today. As an example, the prevalence of methicillin-resistant *Staphylococcus aureus* (MRSA) increased from 5% in 1980 to 50% of documented *S. aureus* infections in 2000^1^. The increase in AMR is placing significant pressure on essential health care systems around the world^2^ as infections caused by resistant bacteria are associated with higher levels of morbidity and mortality and increased health care costs^3^. Furthermore, in 2016 more than 2800 people died from infectious diseases in Australia^4^, highlighting the need for effective antibiotic therapy as a critical component of reducing both morbidity and mortality associated with infectious diseases.

Naturally-occurring stilbenes are known for their diverse bioactivities^5^ including anti-cancer (phoyunbenes^6^, halophilol A^7^ and combretastatins^8^), anti-diabetic (rumexoid^9^) and antimalarial activity (stilbenes isolated from *Artocarpus integer*^10^). Many stilbene derivatives have also shown activity against bacteria^11^ including a compound isolated from *Combretum woodii* leaves^12^.

**Figure 1:**
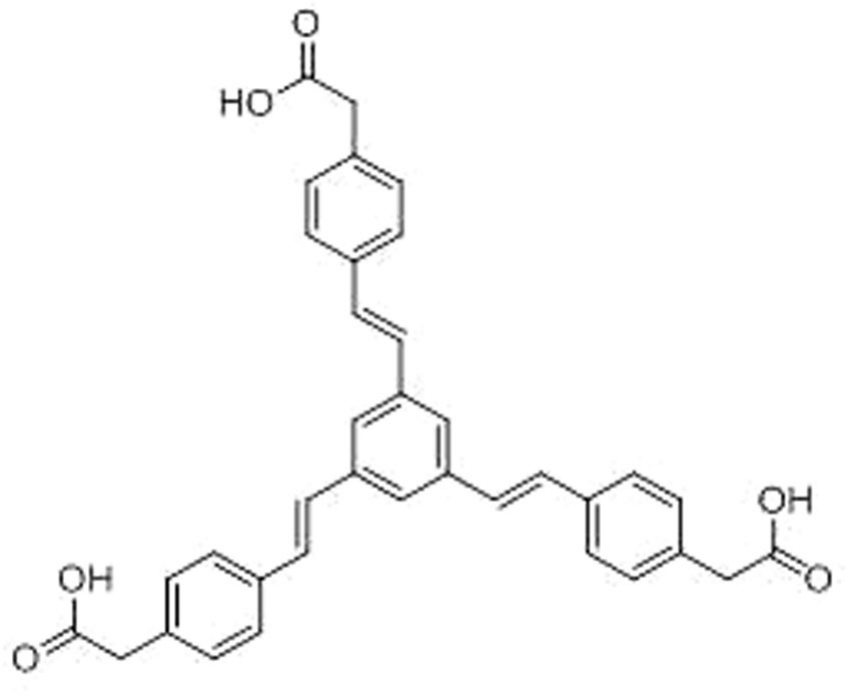
Structure of compound **135C**.

A previous study of a promising antibacterial stilbene compound found *in silico* led to triacid derivative **135C** (Fig. 1). In this previous medicinal chemistry study, **135C** was found to exhibit antibacterial activity^13^ against several Gram-positive bacteria *in vitro*, including methicillin-resistant *Staphylococcus aureus* (MRSA), at concentrations comparable to those required for existing antibiotics^14^. Our reference compound **135C** contains a tri-carboxylic acid functionality and a tri-styrene motif, and its structural class is not seen in the current antimicrobial literature. Other natural stilbenes with antibacterial activity, such as resveratrol^15^, have been studied in detail but none contain this tri-styrene motif. Thus, given that this compound falls outside any known existing antibacterial classes, the benefits from developing it as a potential antibacterial agent are considerable, as there may be a reduced likelihood of observing antibacterial cross-resistance^16^ and it is possible that the compound has a novel mode of antibacterial action.

The aim of the current research was to continue investigations into the antibacterial activity of compound **135C**. In particular, the spectrum of activity against a wide range of organisms was determined and the mode of action was investigated. The spectrum of antibacterial activity and synergy with conventional antibacterial agents was determined using broth microdilution techniques. Detection of the leakage of intracellular material that absorbs at 260 nm, lysis of cells and time-kill experiments were also performed to investigate potential modes of action. Lastly, the development of multi-step resistance to **135C** was investigated by serial passage using four *S. aureus* strains. A genomic comparison of the resultant **135C**-resistant mutant strains and wildtype parent strains was also performed.

## Materials and Methods

### Bacterial strains

Reference and clinical strains (n = 93) were obtained from the School of Biomedical Sciences at The University of Western Australia (UWA) and PathWest Laboratory Medicine WA. The Gram positive isolates (n = 71) comprised staphylococci (n = 40), streptococci (n = 20), micrococci (n = 10) and *Clostridium difficile* (n = 1). The Gram negative isolates (n = 22) included *Moraxella* spp. (n = 10), *Escherichia coli* (n = 3), *Pseudomonas aeruginosa* (n = 3), *Prevotella bivia* (n = 1) and *Bacteroides* spp. (n = 5).

### Compounds

Compound **135C** was synthesised as described previously^14^ and purity was confirmed by ^1^H and ^13^C NMR spectroscopy. Compound **135C** was a yellow solid, and was stored under argon and protected from light at −20 °C. Stock solutions of **135C** in dimethyl sulfoxide (DMSO) were prepared fresh on each testing occasion and were diluted as required. The maximum concentration of DMSO present in bioactivity assays was 10%.

### Susceptibility tests

Minimum inhibitory concentrations (MICs) and minimum bactericidal concentrations (MBCs) were determined using the broth microdilution methods recommended by the Clinical and Laboratory Standards Institute^17^. Briefly, compound **135C** was serially diluted two-fold in 100 µL volumes of sterile distilled water in a 96-well microtitre tray (Falcon, Becton Dickinson, USA). Inocula were prepared by culturing organisms overnight on blood agar, then suspending several colonies in 0.85% saline. Each suspension was then adjusted using 0.85% saline to a 0.5 McFarland turbidity standard. Suspensions were then diluted in Mueller-Hinton broth to give a cell concentration of approximately 10^6^ CFU/ml. Each well of the microtitre tray was inoculated with a 100 µL volume of inocula and microtitre trays were incubated at 37 °C for 24 h, or 48 h for anaerobes, under the appropriate conditions. For a subset of Gram-negative bacteria, MICs were also determined in the presence of polymyxin B nonapeptide (PMBN) at a final concentration of 5 μg/ml. PMBN is a well-known outer membrane permeabiliser^18^ and increased susceptibility in the presence of PMBN suggests that the Gram negative outer membrane has a role in preventing the entry of antimicrobial compounds in the cell^19^. MICs were determined as described above with the following adjustment: **135C** was serially diluted two-fold in 50 μL volumes in a 96-well microtitre tray (Falcon, Becton Dickinson, USA), after which 50 μL of PMBN at 20 μg/ml was added to each microtitre well. Novobiocin (0.5–512 μg/ml) was used as a positive control for assays with PMBN^20^. For all susceptibility tests, MICs were determined visually as the lowest concentration of antibacterial agent preventing visible growth. For some assays, MBCs were determined by subculturing 10 µL volumes from non-turbid wells, spot-inoculating onto blood agar and incubating for 24–48 h. The MBC was determined as the lowest concentration of antibacterial with no resultant growth in the subculture. MBCs were not determined for anaerobic bacteria nor the assay with PMBN. Each organism was tested in triplicate and modal values were selected. DMSO was tested in parallel as a solvent control.

### Determination of antibacterial synergy

MICs of **135C** in combination with a range of antibiotics with varying mechanisms of action were determined against *S. aureus* NCTC 6571 to investigate whether synergy or antagonism occurred. The antibiotics tested were ciprofloxacin, rifampicin, vancomycin, erythromycin, gentamicin and oxacillin. The checkerboard broth microdilution method was used as described previously^21^. Briefly, each antibiotic was serially diluted two-fold in 50 μL volumes in a 96-well microtitre tray (Falcon, Becton Dickinson, USA), after which 50 μL of **135C** at varying concentrations (ranging from 0.06–4 μg/ml) was added to each dilution series. The remainder of the assay was performed as described above. For each well of the microtitre plate that corresponded to an MIC, the fractional inhibitory concentration index (∑FIC) was determined using the equation: ∑FIC = FIC_A_ + FIC_B_ = (MIC of drug A in combination/MIC of drug A alone) + (MIC of drug B in combination/MIC of drug B alone). The ∑FIC_min_ and ∑FIC_max_ were determined as the lowest and the highest ∑FIC, respectively. The interaction was deemed synergistic if the ∑FIC was ≤ 0.5, additive if the ∑FIC was between 0.5 and 1.0, indifferent if the ∑FIC was between 1.0 and 2.0 and antagonistic if the ∑FIC was > 2.0^22,23^.This assay was performed in triplicate per antibacterial agent/**135C** combination.

### Cellular leakage and lysis

Bacteriolysis and cell leakage assays were performed as previously described^24^. Inocula were prepared from an overnight culture of *S. aureus* NCTC 6571 in Mueller-Hinton broth (MHB) by diluting to 0.5 McFarland in fresh MHB. Bacterial suspensions were then left untreated (untreated control) or were treated with 2, 32 (1 × MBC) and 320 μg/ml (10 × MBC) **135C**. Treatments and the control were incubated at 35 °C with shaking at 80 rpm. Samples were removed immediately after inoculation (time 0), and further samples were taken at 15, 30, 60, 120 and 240 min. Cell lysis was determined by measuring the optical density (OD) of each sample at 600 nm. The leakage of intracellular contents was determined by filtering samples (0.45 μm) to obtain cell-free filtrates and determining the OD_260_ at time zero and 240 min only. The OD of all samples was determined against the appropriate blank at the appropriate wavelength. Each assay was performed in triplicate and mean values were determined.

### Time-kill kinetics

Bacterial killing assays were performed as previously described^24^. Inocula were prepared as described for the cell leakage and lysis assays and were exposed to **135C** at 0 (untreated control), 32 and 320 μg/ml of **135C** in MHB. Treatments and controls were incubated at 35 °C with shaking at 80 rpm and samples were removed at 0, 15, 30, 60, 120 and 240 min. Samples were immediately serially diluted ten-fold in 0.01 M phosphate buffered saline (PBS) at pH 7.0 and 10 μL aliquots were spot-inoculated in duplicate onto MHA. Agar plates were incubated at 37 °C overnight before determining the viable count. The assay was conducted on two separate occasions.

### Serial passage with compound **135C** to evaluate resistance development

Four *S. aureus* strains (NCTC 10442, ATCC 29213 and clinical isolates RPH 15913 and 3784546E) were investigated for the development of multi-step resistance to **135C** by serially passaging in increasing concentrations of the compound, using methods as previously described^25^. Briefly, MICs were determined using the CLSI broth microdilution method as described above^17^. The optical density of all wells was then determined (at 600 nm) and serial passage was performed by removing the contents of the well with the highest concentration of **135C** that had an OD_600_ value of approximately 0.1, and adding this culture to a freshly prepared dilution series in a 96-well microtitre tray. This was repeated at 24 h intervals for a total of 10 passages, for each of the four organisms. The compound concentration range of each new passage was based on the MIC from the previous passage. At the end of the serial passage experiment, each organism was cultured from the highest turbid well onto blood agar (BA) and all were then passaged daily on drug-free media (BA) for 10 days. MICs of **135C** were determined again after the third and tenth drug-free passages. After three drug-free passages, MICs were also determined for ciprofloxacin, erythromycin, gentamicin, oxacillin, rifampin, vancomycin, kanamycin and chloramphenicol to evaluate whether antibacterial cross-resistance was evident. The entire experiment was performed four times.

### Relative fitness of *S. aureus* isolates serially passaged with 135C

The relative fitness of strains was evaluated as described previously^26^. Briefly, serially-passaged *S. aureus* isolates and parent strains were cultured overnight on brain heart infusion agar (BHIA) plates, then 2–3 well-isolated colonies were inoculated into 15 ml of brain heart infusion broth (BHIB) and incubated at 37 °C with shaking at 80 rpm. After overnight growth, all cultures were diluted to an OD_600_ of 0.01 and 150 μl of this was added to 15 ml of BHIB to give an inoculum concentration of approximately 10^6^ CFU/ml. Cultures were incubated at 37 °C with shaking at 125 rpm for 24 h. The OD_600_ of cultures was determined every hour for 1–7 h and then at 24 h. The entire assay was performed four times.

### Genomic DNA preparation and whole genome sequencing

Following subculture of *S. aureus* isolates on blood agar for 24 h, genomic DNA was extracted using a Gentra Puregene Yeast/Bact. Kit [Qiagen, Hilden, Germany] and multiplexed paired-end (PE) libraries were generated using standard Nextera XT protocols [Illumina, CA, USA]. Whole genome sequencing (WGS) of eight *S. aureus* strains (4 generated mutants, 4 wildtype) was performed using a MiSeq benchtop sequencer [Illumina], generating 300 bp PE reads. Sequence data for this study has been deposited in the European Nucleotide Archive (ENA) under study PRJEB21492 (sample accessions ERS1797685-ERS1797692). To examine genetic differences between wildtype parent strains and mutant progenitors, high-resolution single nucleotide variant (SNV) analysis was performed, as previously described^27^. The highly annotated genome of *S. aureus* strain MRSA-252 [Genbank accession BX571856, multilocus sequence type (ST) 36] was used as a reference chromosome for read mapping (to a median depth of 45X across the 2.9 Mbp chromosome, range 26.9–52.6X) and SNV calling^28^.

## Results

### Susceptibility tests

Susceptibility testing of a range of aerobic bacterial species showed that compound **135C** was active against Gram-positive bacteria and also active against the Gram negative species *Moraxella catarrhalis* (Table 1). The most susceptible organism was *S. aureus*, with MICs of **135C** ranging from 0.12–0.5 μg/ml. However, the MBCs for all species tested were ≥ 32 μg/ml (Table 1). Given this large difference between the MICs and MBCs, the activity of **135C** was deemed to be bacteriostatic. When MICs against *S. aureus* NCTC 6571 were determined under anaerobic conditions, the MIC decreased from 0.5 to 0.004 μg/ml (seven doubling-dilutions). Susceptibility tests against a range of anaerobic bacteria showed an MIC of 16 µg/ml for *Clostridium difficile*, and a range of 32–256 µg/ml for *Prevotella bivia* and five *Bacteroides* spp. strains.

**Table 1:** Minimum inhibitory and bactericidal concentrations of **135C**.

Organism	n	MIC (µg/ml)			MBC (µg/ml)
		Range	MIC_50_	MIC_90_	
*Micrococcus* spp.	10	8–>32	16	>32	>32
*Moraxella catarrhalis*	10	1–>32	8	32	>32
*S. aureus* (methicillin resistant)	10	0.12–0.5	0.25	0.5	32–256
*S. aureus* (methicillin susceptible)	10	0.25–0.5	0.25	0.5	32–128
*Staphylococcus epidermidis*	10	1–32	2	4	>32
*Staphylococcus* spp. (CNS)^1^	10	0.5–>32	4	>32	>32
*Streptococcus pneumoniae*	10	1–16	4	16	>32
*Streptococcus pyogenes*	10	8–32	8	16	>32

^1^CNS, coagulase-negative = *Staphylococcus hominis*, *S. warneri*, *S. saprophyticus*, *S. capitis* and *S. haemolyticus*.

### MICs in the presence of PMBN

The activity of **135C** against *E. coli* and *P. aeruginosa* was enhanced in the presence of PMBN (Table 2). The effect was more pronounced for *E. coli* than for *P. aeruginosa*, with the prior exhibiting at least a three-doubling-dilution increase in susceptibility to **135C** in the presence of PMBN.

**Table 2:** Minimum inhibitory concentrations of **135C** for Gram-negative bacteria in the presence and absence of PMBN.

Organism	MIC (µg/ml)			
	135C alone	135C+PMBN	Novobiocin alone	Novobiocin+PMBN
*E. coli* ATCC 43889	>512	128	64	2
*E. coli* NCTC 10538	>512	32	256	4
*E. coli* ATCC 25922	>512	128	128	2
*P. aeruginosa* NCTC 6749	512	256	>512	n.d.
*P. aeruginosa* ATCC 27853	512	n.d.	256	1
*P. aeruginosa* ATCC 25668	512	512	512	8

n.d. = no data.

PMBN concentration 5 μg ml^−1^.

### Synergy experiments

**Figure 2:**
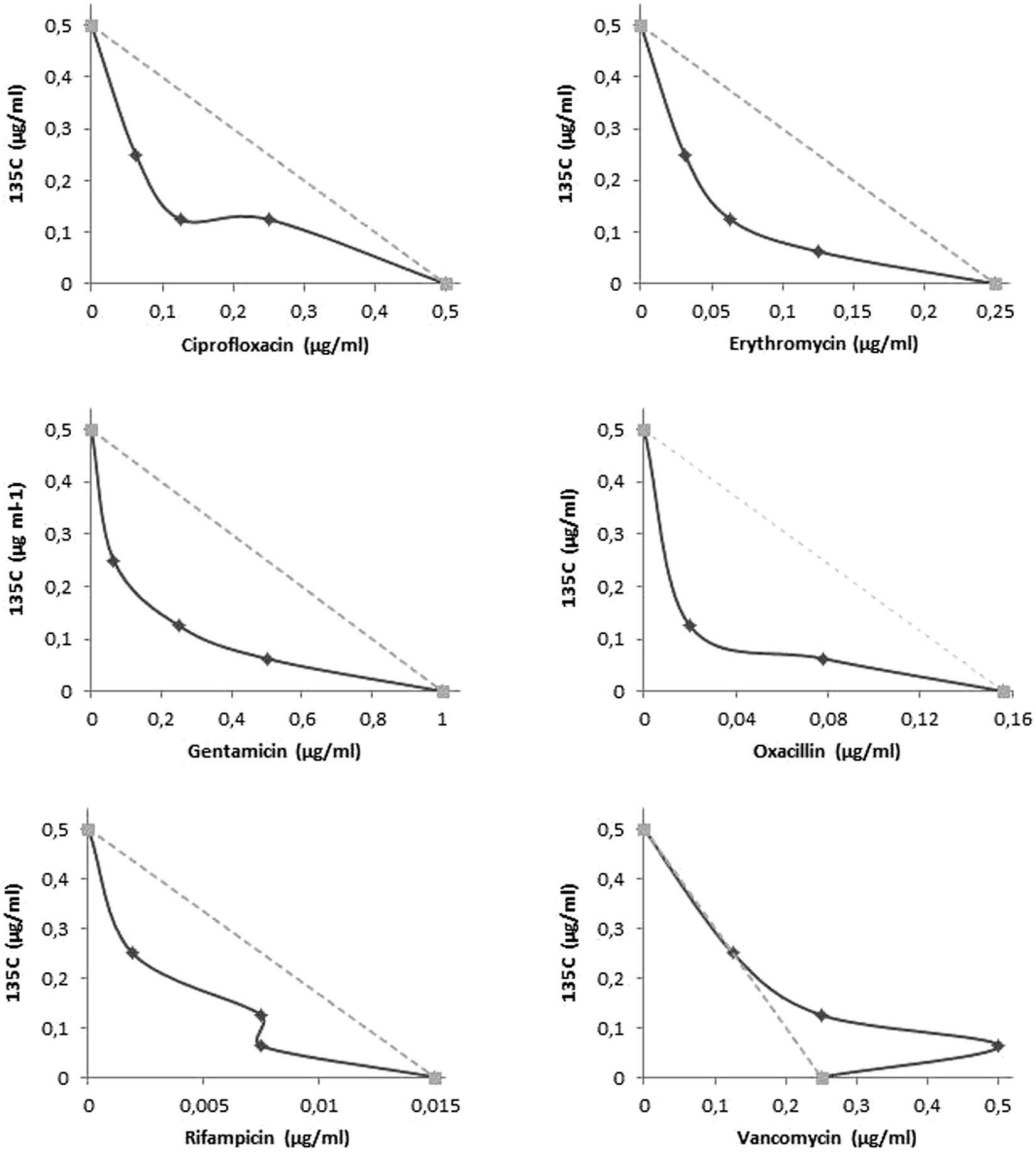
Isobolograms showing interactions between **135C** and antibacterial agents. The dotted gradient line represents the theoretical line of additivity.

Antibacterial synergy data were generated using oxacillin, gentamicin, erythromycin, vancomycin, rifampicin and ciprofloxacin (Fig. 2). The ∑FIC was calculated to determine whether each combination of antibacterial agent/**135C** was considered synergistic, additive, indifferent or antagonistic (Table 3). Although there were several ∑FIC_min_ values that were less than 0.5, the results overall showed largely additive and indifferent activity for all six antibacterial agents, indicating negligible synergistic activity with **135C**.

**Table 3:** Minimum and maximum Fractional Inhibitory Concentration (FIC) indices of antibacterial agent/**135C** combinations.

Antibiotic	FIC_min_	FIC_max_	Interaction
Ciprofloxacin	0.5	1.125	Additive
Erythromycin	0.376	1.125	Additive
Gentamicin	0.375	1.125	Additive
Oxacillin	0.531	1.015	Additive
Rifampicin	0.375	1.125	Additive
Vancomycin	1	2.25	Indifferent

### Leakage, lysis and time-kill experiments

Treatment of *S. aureus* NCTC 6571 with the highest concentration compound **135C** (320 μg/ml, 10 × MBC) did not lead to any decrease in OD_600_, indicating that cell lysis did not occur. Similarly, experiments investigating whether exposure to **135C** resulted in the leakage of intracellular contents did not show an appreciable change in OD_260_ after 2 h, indicating that the leakage of intracellular materials (nucleic acids) did not occur and that the cell membrane likely remained intact. Time-kill experiments with *S. aureus* NCTC 6571 did not show cell death at any concentration of **135C**, with bacterial growth occurring in the presence of 2 µg/ml and 32 µg/ml similar to the drug-free control. At 320 μg/ml (10 × MBC), cell viable counts remained similar to time zero, indicating that growth was inhibited but that cell death did not occur under these conditions.

### Multi-step serial passage of *S. aureus* isolates with compound **135C**

**Figure 3:**
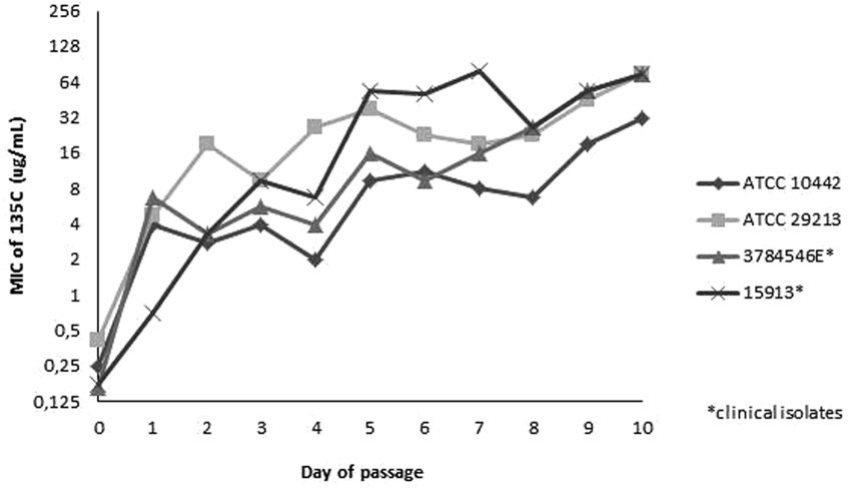
Serial passage of *S. aureus* strains with **135C** showing MICs over time. Data points are the geometric mean of two independent experiments repeats, each conducted in duplicate (4 values per day per isolate).

**Figure 4:**
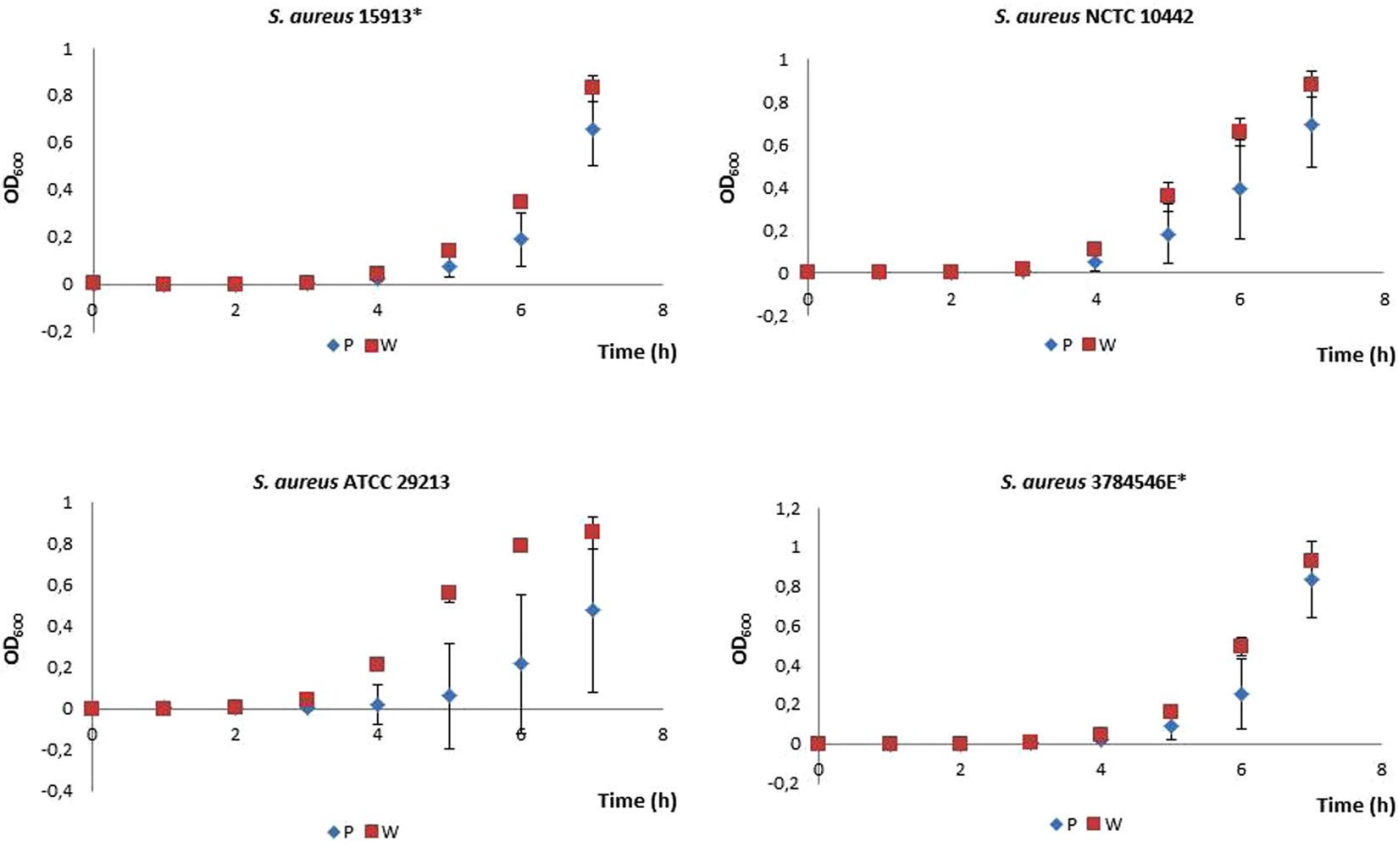
Growth fitness of serially passaged *S. aureus* isolates. The W indicates the wild-type strain and P indicates the passaged strain.

Four *S. aureus* isolates were serially passaged with increasing concentrations of compound **135C** to investigate the development of resistance. After the first passage, MIC values for all four isolates had increased by at least 3-fold (Fig. 3) After ten passages with increasing concentrations of compound **135C**, MICs for all strains had increased from initial values of 0.12–0.25 μg/ml to 32–64 μg/ml. To investigate whether resistance was stable, isolates were subsequently passaged 10 times without **135C**, and MICs were again determined. For all four strains, the MICs after 10 drug-free passages were 32 μg/ml, which is similar to the final values (32–64 μg/ml) obtained after the 10 serial passages. This indicates that changes in susceptibility were stable, and are possibly due to a defined genetic mutation. The susceptibility of serially passaged *S. aureus* isolates was determined for a range of antibacterial agents selected to cover five major mode of action targets for antibiotics within bacterial cells. Cross-resistance to an antibacterial agent could indicate a shared, or at least similar, mode of antibacterial action^16^. However, parent and serially passaged resistant strains did not differ in susceptibility (Table 4). Therefore, this part of the study did not offer any new information on the mode of action of compound **135C**. Growth experiments conducted with both wildtype and serially-passaged **135C**-resistant strains (Fig. 4) showed minor differences in growth patterns, with the greatest difference seen for *S. aureus* ATCC 29213.

**Table 4:** Evaluation of *S. aureus* isolates passaged with **135C** for cross-resistance to antibacterial agents.

Organism	Strain	MIC (µg/ml)								
		CIP	ERY	GEN	OXA	RIF	VAN	KAN	CHL	135C
*S. aureus* NCTC 10442	Parent	1	1	2	>16	0.0078	1	8	8	0.25
	135C-passaged	0.5	1	1	>16	0.0078	1	4	8	>8
*S. aureus* ATCC 29213	Parent	1	2	0.5	0.25	0.006	1	8	16	0.5
	135C-passaged	0.5	1	1	0.5	0.0078	1	4	16	>8
*S. aureus* 15913*	Parent	>8	>16	>16	>16	0.0078	1	>128	16	0.25
	135C-passaged	>8	>16	>16	>16	0.0039	2	>128	8	>8
*S. aureus* 3784546E*	Parent	>8	>16	2	>16	0.0078	1	8	16	0.5
	135C-passaged	>8	>16	2	>16	0.0078	1	4	16	>8

CHL, chloramphenicol; CIP, ciprofloxacin; ERY, erythromycin; GEN, gentamicin; KAN, kanamycin; OXA, oxacillin; RIF, rifampin; VAN, vancomycin.

*Clinical isolate.

### Genomic comparison of generated mutants and wildtypes

High-quality SNVs were detected in all four *S. aureus* mutant strains, relative to their wildtype counterparts (Table 5). Three of the four **135C**-resistant strains showed nonsynonymous nucleotide changes (point mutations) in genes associated with teichoic acid biosynthesis (*tagH*, *tagA*, and *tagG*). These missense mutations resulted in codon changes and non-synonymous amino acid changes in the respective tag proteins.

**Table 5:** Analysis of heterozygous sites in wildtype and mutant strains of *S. aureus*.

Strain	Position	WT	MUT	Locus tag	Gene	Variant Strand^†^	Protein change	Product description
*S. aureus* 3784546E*	551633	C	T	SAR0512	*ftsH*	—	—	Cell division protease FtsH
	692985	T	C	SAR0647	*tagH*	A757G (minus)	p.Arg253Gly	Teichoic acid ABC transporter ATP-binding protein
*S. aureus* NCTC 10442	692787	G	T	SAR0646	*tagA*	G665T (plus)	p.Arg222Ile	Teichoic acid biosynthesis protein
*S. aureus* RPH 15913*	694216	C	G	SAR0648	*tagG*	C129G (plus)	p.Asn50Lys	Teichoic acid ABC transporter permease protein
*S. aureus* ATCC 29213	838911	G	A	SAR0801	—	—	—	Putative glycosyl transferase
	1721885	C	T	SAR1654	—	—	—	Conserved hypothetical protein (methyltransferase)
	2506091	C	T	SAR2437	—	—	—	Putative transport protein

WT, wildtype.

MUT, mutant.

MRSA-252 Genbank accession BX571856.

*Clinical isolates.

p. denotes amino acid change.

^†^Base pair change on forward (plus) or reverse (minus) DNA strand.

## Discussion

The current study showed that the novel compound **135C** was active largely against Gram-positive bacteria and confirmed the lack of activity observed previously against Gram-negative bacterial species^14^. Compound **135C** showed highest activity against *S. aureus*, and was slightly less active against other Gram positive organisms, for reasons that remain to be determined. Poor activity against most Gram-negative bacteria may be due to a number of mechanisms including outer membrane impermeability and active efflux systems^20^. It has been shown previously that the outer membrane permeabiliser PMBN interacts with the lipopolysaccharide of the outer membrane of Gram-negative bacteria, and PMBN has been used to sensitise different bacteria to many hydrophobic antibacterial agents such as novobiocin, erythromycin and clindamycin^29^. PMBN alone has negligible activity and does not cause the leakage of periplasmic proteins from bacteria^30^ but allows the entry of hydrophobic antibacterial compounds into the cell by facilitating hydrophobic diffusion through the outer membrane. The activity of **135C** was enhanced in the presence of PMBN, suggesting that the outer membrane has a role in limiting or preventing the entry of this compound into the cell. Further evidence of outer membrane involvement is shown by the susceptibility of the unusual Gram negative *M. catarrhalis* to **135C**, which is likely due to the lack of long O-antigen polysaccharide chains in the lipopolysaccharide structure of its outer membrane^31^, which are normally present in Gram-negative bacteria. The absence of these lipooligosaccharide chains results in the increased permeability of the bacterial envelope^31^, which may contribute to the susceptibility of this organism to **135C**.

When *S. aureus* species switch from aerobic to anaerobic growth, changes in gene expression occur, for example the presence of glycolytic enzymes, and comparatively low amounts of tricarboxylic acid cycle enzymes^32^. The MIC of **135C** against *S. aureus* NCTC 6571 decreased from 0.5 μg/ml to 0.004 μg/ml under anaerobic conditions. This suggests that under such conditions, the compound may be oxidised to a less active derivative, or that the compound may be metabolised differently under anaerobic conditions.

The mode of action of **135C** against *S. aureus* was investigated using several different approaches. The first approach was to investigate effects against the cell wall and cell membrane, as these are two major targets for current antibacterial agents^33^ and experiments to quantify one or both are often performed to elucidate the mode of action of novel antibacterial agent^24^. Unfortunately, the results from the cell leakage and lysis assays indicated that neither of these sites are likely to be major targets for **135C**.

Subsequently, studies investigating antibacterial synergy were conducted. Synergy between different antibacterial compounds can be determined using the checkerboard method where multiple concentrations of two different compounds are tested in combination. There are many examples of antibacterial combinations that have been shown to be synergistic, and synergistic interactions have the potential to shed light on the mode of action of an antibacterial agent. Examples include compounds that are active against the cell wall enhancing the uptake of aminoglycosides, as well as combinations of agents active on the cell wall^34^. Compound **135C** was tested in combination with antibacterial agents from different classes with known modes of action to broadly identify which class **135C** may fall into. Antibacterial synergy data was determined using oxacillin, gentamicin, erythromycin, vancomycin, rifampicin and ciprofloxacin. The results showed only additive effects for all six antibacterial agents and did not show any observable significant synergy nor antagonism (Fig. 2).

Investigation of the development of resistance to **135C** by *S. aureus* isolates showed rapid and stable resistance, which limits the potential usefulness of the compound. Often, the acquisition of bacterial resistance is accompanied by a fitness cost to bacteria^35–37^, typically evident as a reduction in growth rates *in vivo*^38^ and *in vitro*^39^, decreased invasiveness^40^ or a lower cell density^41^. Data from the current study indicated only minor differences in the fitness of parent and serially passaged strains, suggesting that fitness was not substantially altered.

Genomic comparison of the four **135C**-resistant mutants to their wildtype parent strains provided interesting insight into a potential relationship between **135C** and wall teichoic acids (WTAs) (Table 5). Of the four **135C**-resistant strains, three contain mutations within the teichoic acid glycerol (tag) genes, *tagH*, *tagA*, and *tagG*. These three genes are involved in protein products associated with teichoic acid biosynthesis (*tagA*), and their ATP-binding cassette (ABC) transporter subunits (*tagH* and *tagG*)^42–44^. The changes identified in the **135C**-resistant mutants suggest that WTAs may be the target site of **135C**, or that the compound may disruption of the regular function of WTAs. This finding is also helps to explain the lack of activity against Gram negative bacteria, as teichoic acids are absent in Gram negative bacteria.

Of note was that the changes in all three *tag* genes were nonsynonymous substitutions. These missense mutations (single nucleotide alterations) result in codon changes that lead to different amino acids^45^. These changes occurred in only three of the four strains, with these three strains all being methicillin-resistant whilst the fourth strain is methicillin susceptible. The amino acid changes in *S. aureus* 3784546E and NCTC 10442 both involved an arginine substitution. In both cases, the substitution led to amino acids with shorter (less flexible), more hydrophobic and non-polar side chains (glycine and isoleucine)^46^. For *S. aureus* RPH 15913, the mutation resulted in an amino acid change from asparagine to lysine. The lysine side chain contains a longer (more flexible) and less hydrophobic moiety than asparagine, which is in contrast to what was observed in the previous two strains. However, for all three strains the mutation resulted in a less polar amino acid^47^. The polarity of the amino acid changes may be a significant factor if WTAs are the binding sites of **135C**. Structurally, **135C** is a symmetrical molecule with three polar carboxylic acid moieties^14^. It is therefore plausible that a change in the polarity of an amino acid in WTAs may have an effect on the ability of the acid moieties within this compound to hydrogen bond to a potential binding site^48,49^. Therefore, from a structural perspective, the changes in the amino acid side chains may be significant if the *tag* genes are indeed the binding sites of **135C**.

It was interesting to note that *S. aureus* ATCC 29213 (methicillin-susceptible) was the only strain without a change in a *tag* gene. A plausible explanation may be that this strains’ WTA genetic profile is different to that of the methicillin-resistant strains. The mode of action of the *β*-lactam antibiotic methicillin involves the inhibition of penicillin-binding proteins (PBPs), which are essential in the synthesis of the peptidoglycan layer that makes up the cell wall^50^. Since WTAs are embedded in the peptidoglycan layer, the resistance mechanism of MRSA may have also affected these sites^44,51^. It is also important to note that WTAs are classified as virulence factors^42,52^, and as such, **135C** may have a downstream effect on the ability of Gram-positive bacteria to proliferate in host organisms.

To summarise, our study revealed **135C** to be a bacteriostatic agent with activity against a range of Gram-positive pathogens. The compound was largely inactive against Gram-negative bacteria, which may be due, in part, to the impermeability of the Gram-negative outer membrane and absence of teichoic acids within the cell wall. When combined with conventional antibiotics **135C** did not show significant synergistic nor antagonistic activity. The absence of leakage of bacterial cell contents and absence of cell lysis indicate that **135C** is unlikely to have a target within these sites. Resistance studies showed that *S. aureus* rapidly developed stable resistance to **135C**, after as little as a single passage. Comparison of the whole genome sequence of the **135C**-resistant mutants to their wildtype counterparts revealed a change in teichoic acid-associated *tag* genes. Changes in these genes indicate a possible interaction between **135C** and WTAs as part of its mode of action. Additional studies with bacterial strains that have specific WTA mutations^53–55^ may help to clarify the role of WTA in susceptibility to **135C**. This may also offer insight into why *S. aureus* in particular, is highly susceptible compared to other Gram positive organisms. In conclusion, lack of activity against Gram negative pathogens, absence of bactericidal activity and the rapid development of resistance all significantly limit the potential usefulness of the compound. It remains possible that additional chemical modification of the compound may lead to further novel compounds with improved efficacy, broader spectrum of activity and reduced resistance capacity.
